# The Role of Emotional Understanding in Academic Achievement: Exploring Developmental Paths in Secondary School

**DOI:** 10.3390/jintelligence13080096

**Published:** 2025-07-30

**Authors:** Luísa Faria, Ana Costa, Vladimir Taksic

**Affiliations:** 1 Centre for Psychology at University of Porto (CPUP), Faculty of Psychology and Education Sciences, University of Porto, Rua Alfredo Allen, 4200-135 Porto, Portugal; 2 Center for Philosophical and Humanistic Studies (CEFH), Faculty of Philosophy and Social Sciences, Portuguese Catholic University, Praça da Faculdade de Filosofia 1, 4710-297 Braga, Portugal; ancosta@ucp.pt; 3 Department of Psychology, Faculty of Humanities and Social Sciences, University of Rijeka, Sveučilišna Avenija 4, 51000 Rijeka, Croatia; vtaksic@ffri.uniri.hr

**Keywords:** emotional understanding, ability-based emotional intelligence, academic achievement, secondary education, Latent Growth Curve (LGC)

## Abstract

The role of emotional intelligence (EI) in the academic context has been steadily established, together with its impact on students’ academic achievement, well-being, and professional success. Therefore, this study examined the development of a key EI ability—emotional understanding—throughout secondary school and explored its impact on students’ academic achievement (maternal language and mathematics) at the end of this cycle, using the Vocabulary of Emotions Test. A total of 222 students were followed over the entire 3-year secondary cycle, using a three-wave longitudinal design spanning from 10th to 12th grade. At the first wave, participants were aged between 14 and 18 years (M = 15.4; SD = 0.63), with 58.6% being female. Overall, the results of Latent Growth Curve modeling indicated that students’ emotional understanding increased over the secondary school cycle. While student’s gender predicted the emotional understanding change patterns throughout secondary school, student’s GPA in 10th grade did not. Moreover, the initial levels of ability-based emotional understanding predicted students’ achievement in maternal language at the end of the cycle. Our findings offer valuable insights into how EI skills can contribute to academic endeavors in late adolescence and will explore their impact on educational settings.

## 1. Introduction

In the last few decades, psychology has made significant contributions by highlighting the role of emotional and motivational factors as crucial predictors of success in both school and life, influencing individuals’ adaptation, well-being, and overall success (Brackett et al. 2016; Caballero-García and Ruiz 2025; Doǧru 2022; Joseph et al. 2015; Llamas-Díaz et al. 2022; MacCann et al. 2020; Van Rooy and Viswesvaran 2004). In the academic context, research has shifted from concentrating primarily on cognitive predictors to a broader understanding of academic success as a multidimensional concept (Araújo 2017; Costa et al. 2024; Green et al. 2012; Quílez-Robres et al. 2021).

Indeed, students’ academic and emotional experiences, as well as their coping strategies and resources, play a significant role in shaping their personal, academic, and professional trajectories. In this regard, existing research underlines emotional intelligence (EI) abilities—including perceiving and expressing emotions, using emotions to facilitate thought, understanding emotions, and regulating emotions in oneself and others (Mayer and Salovey 1997)—as key factors influencing both emotional and academic outcomes. Studies have shown that effectively managing emotions is vital for improving mental and physical well-being (Barchard and Houghton 2022; Schutte et al. 2007), while also enhancing performance in academic settings (MacCann et al. 2020).

Adolescence involves complex decision-making and emotional self-regulation competencies as adolescents assume greater responsibilities at home, school, and in social relationships. Additionally, this period is marked by concerns about personal and academic achievement, as well as stress from physical and self-image transformations (Somerville 2016), while their self-perceived ability, school engagement, and academic performance often decline (Fredricks et al. 2004; Sundblad et al. 2008; Watt 2004). This developmental stage offers both challenges and opportunities for youth development (Crockett and Silbereisen 2000; Sawyer et al. 2018), where emotional abilities and self-perceptions are key factors in determining their outcomes.

Also, the literature has established that EI abilities can be developed, cultivated, and enhanced over time with training, committed effort, and opportunities for experience (Costa and Faria 2016; Greenberg 2022; Mahoney et al. 2018; Mattingly and Kraiger 2019), which are worthy of investigation due to their potential in enhancing or undermining students’ adjustment, well-being, and achievement. Building on this, the authors seek to investigate how the development of students’ emotional understanding ability over the course of the secondary school influences their academic achievement, highlighting its relevance for both emotional and academic development.

### 1.1. Emotional Intelligence: Conceptualization and Assessment

The recognition of EI’s impact on a wide range of personal outcomes and achievements were clearly established (Brackett et al. 2016): meta-analyses have shown that individuals with higher emotional intelligence (EI) tend to experience better health, well-being, and interpersonal relationships (Andrei and Petrides 2013; Costa et al. 2014; Fernández-Berrocal and Extremera 2016; Kotsou et al. 2011; Llamas-Díaz et al. 2023; Martins et al. 2010; Schutte et al. 2007), while also performing better in academic and professional environments (Caballero-García and Ruiz 2025; Joseph et al. 2015; Joseph and Newman 2010; O’Boyle et al. 2011).

Research in the field has provided substantial evidence leading to an established consensus regarding the two main theoretical orientations of EI. Mixed or trait models of EI incorporate a broad array of components, including not only emotional abilities but also personality traits and motivational factors (Bar-On 2006; Petrides et al. 2007). In contrast, ability-based models define EI as a form of cognitive intelligence, like verbal or numerical reasoning, but specifically focused on the processing and understanding of emotional information (MacCann et al. 2014). In this last perspective, Mayer and Salovey’s pioneering authors (1997) argue that EI consists of distinct yet interconnected cognitive abilities, which together form the four-branch or ability model of EI. This model includes the capacity to accurately perceive, evaluate, and express emotions; to access or generate emotions that facilitate thought; to understand emotional meanings and knowledge; and to regulate emotions in ways that foster both emotional and cognitive growth (Mayer and Salovey 1997).

In a parallel way, scholars have emphasized the importance of differentiating between ability and mixed EI’s distinct types of assessment tools (Mayer et al. 2000). Ability-based tests assess emotional intelligence by requiring individuals to solve problems involving emotional information, thereby reflecting their actual ability in this domain. On the other hand, self-report questionnaires ask individuals to evaluate their own emotional skills by indicating their level of agreement with various personal statements (Brackett et al. 2006). Although significant progress has been made in the conceptualization and assessment of EI, ongoing efforts are still needed to refine existing instruments and develop new psychometrically sound measures capable of capturing the full complexity of the construct.

### 1.2. Emotional Intelligence and Academic Achievement

Academic achievement research was initially linked only to cognitive abilities; nonetheless, it is now well established that both personal and contextual factors contribute to students’ achievement (Costa et al. 2024; MacCann et al. 2020). Students’ academic achievement is often considered a relevant metric for evaluating academic success and a predictor of educational trajectories (Steinmayr et al. 2014), since it is associated with the quality of individuals’ functioning across various settings and over the lifespan (Cachia et al. 2018; Droppert et al. 2019). Academic achievement or performance is usually assessed through exams and various subject-specific assessments conducted during the school year, and it is typically represented by average grades across subjects (such as GPA or SAT scores), which allows for comparison with peers (Richardson et al. 2012).

Emotional intelligence (EI) has been widely investigated within the academic context, with evidence suggesting a positive effect on students’ educational experiences (Caballero-García and Ruiz 2025; Molero et al. 2020; Sánchez-Álvarez et al. 2015). Indeed, students with higher EI levels tend to be more motivated and better at managing autonomous and self-regulated learning (Inglés et al. 2017; Kumar et al. 2013; Nieto et al. 2024; Tam et al. 2021). Additionally, students who effectively regulate their emotions tend to be happier and enjoy stronger social connections, as emotional and interpersonal competence is linked to broader social networks, collaborative learning environments, and higher-quality friendships (Eryilmaz 2011; Fuertes Meza et al. 2023; Sánchez-Álvarez et al. 2015). In the face of challenging situations, EI enables students to cope with academic stress, fosters academic resilience, and reduces negative emotions such as anxiety (Fiorilli et al. 2020), allowing them to maintain or improve performance (Gavín-Chocano et al. 2022; Schneider et al. 2013). Moreover, EI skills align with academic skills more strongly associated with humanities (e.g., history and language arts) due to their focus on understanding human emotions and motivations (MacCann et al. 2020). Supported by the available empirical evidence, it is well accepted that EI contributes to students’ better levels of academic achievement and performance. In MacCann et al.’s (2020) meta-analysis, after controlling intelligence and the Big Five personality traits, EI was found to have additional predictive validity in academic achievement. This effect was stronger for the EI ability model (ρ = 0.24, k = 50) compared to other theoretical approaches (self-rated ρ = 0.12, k = 33; mixed EI ρ = 0.19, k = 90). In particular, the experiential dimensions of understanding and managing emotions appear to play a key role in students’ adjustment to the academic environment. The ability to manage emotions helps students cope with negative feelings related to school performance and fosters more positive interpersonal relationships with both peers and school staff. Furthermore, understanding emotions—including emotional content, the evolution of emotions over time, and how emotions influence thoughts and actions—can be especially relevant for engaging in academic subjects such as history, the arts, and the humanities more broadly (MacCann et al. 2020).

### 1.3. Emotional Intelligence and Demographics

Despite some inconsistent evidence, the assumption that EI increases with age is established (Mayer et al. 1999; Megías-Robles et al. 2024; Salovey and Sluyter 1997). Additionally, there is evidence that EI can be developed (Mattingly and Kraiger 2019) through the accumulation of emotional life experiences, leading to the acquisition of related skills, and new coping strategies to manage emotions more effectively (Brackett et al. 2016; Megías-Robles et al. 2024). In adults, EI can increase with age when assessed either with self-report or performance-based measures (Cabello et al. 2016; Navarro-Bravo et al. 2019). However, some studies have found that both younger and older adults score lower compared to middle-aged adults (Cabello et al. 2016).

For adolescents specifically, the literature is more limited and presents inconsistent findings. While some studies report that EI levels assessed through self-report measures remain stable (Costa and Faria 2016) or even decrease over time (Esnaola et al. 2017; Garaigordobil 2020; Gomez-Baya et al. 2016; Keefer et al. 2013), others have found that ability-based measures show an increase in EI over time (Costa and Faria 2016; Peters et al. 2009). Still, other report negative associations (Fernández-Berrocal et al. 2018) or no significant differences at all (Davis and Humphrey 2012).

Considering gender EI differences, for ability-based measures, females tend to show higher EI levels (Cabello et al. 2016; Palmer et al. 2005) or the absence of significant differences (Costa and Faria 2016). Therefore, gender as a relevant variable that might reflect specific emotional patterns shaped by sociocultural processes or inherent biological differences (Brody and Hall 2008; Chaplin and Aldao 2013) is explored in this study at the beginning of the secondary school cycle, observing its influence on the developmental trajectories of emotional understanding. Students’ academic achievement at the beginning of the secondary cycle will also be considered for controlling specific effects on the explained variance of EI on the end-of-cycle academic outcomes.

### 1.4. The Current Study

As reported previously, EI abilities play a significant role in student’s academic adaptation, well-being, and overall achievement (Gavín-Chocano et al. 2022; MacCann et al. 2020; Nieto et al. 2024; Sánchez-Álvarez et al. 2015; Tam et al. 2021). Nonetheless, research is scarce on student’s EI abilities’ developmental patterns in late adolescence, while their impact on different indicators of students’ academic achievement still needs further clarification. Therefore, this study, employing a longitudinal design and focusing on the emotional ability of understanding emotions, aims to clarify the concurrent role of emotional abilities in shaping students’ academic performance throughout the secondary school cycle. To achieve this goal, the following research questions were proposed: (a) Does the ability to understand emotions evolve throughout the three years of secondary school? (b) Do students’ gender and GPA influence the development of emotional understanding over time? (c) Do the individual trajectories of students’ emotional understanding forecast academic outcomes (mathematics, and maternal language grades) at the conclusion of the secondary school cycle (12th grade)?

## 2. Materials and Methods

### 2.1. Participants

A total of 222 secondary school students (41.4% male) participated in a longitudinal study conducted over the three years of the Portuguese secondary education cycle (grades 10 to 12), involving seven public schools located in a large city in northern Portugal. At the time of the first data collection, participants were aged between 14 and 18 years (M = 15.4; SD = 0.63), with a large portion coming from families with higher socioeconomic status (37.7% high, 28.8% medium, and 33.5% low). Reflecting national patterns (DGEEC 2018), most students were enrolled in science and technology courses (76.1%), while smaller proportions attended languages and humanities (19.4%) and other fields (4.6%). Additionally, most students in this educational cycle typically pursue higher education.

### 2.2. Measures

#### 2.2.1. Vocabulary of Emotions Test (VET)

Vocabulary of Emotions Test (VET)—is a performance-based measure derived from the third branch of the emotional intelligence (EI) model, *Understanding Emotion*. It evaluates emotional knowledge through a vocabulary task focused on words related to emotions. Created by Takšić et al. (2003) for the Croatian academic context with secondary school students and adapted to the Portuguese context by Costa and Faria (2011), the VET consists of 35 items that feature emotionally charged target words. It follows the typical structure of a classic vocabulary test. In the task, participants are asked to select one adjective (out of six options) that best aligns with the meaning of a target emotion word. For instance, given the target word “touching,” the participant must choose the adjective that most closely matches from the following choices: “gentle,” “moving,” “proud,” “sensitive,” “bashful,” or “ruthless.” There is a correct answer based on the dictionary. The VET demonstrated strong psychometric properties (Altaras Dimitrijević et al. 2020; Takšić et al. 2021), including positive correlations with other intelligence tests (California Tests of Mental Maturity—Vocabulary Test: r = 0.67, *p* < .001; Logical Thinking: r = 0.33, *p* < .001), and with other EI tests including the Test of Emotions Understanding (r > 0.50; Altaras Dimitrijević et al. 2020; Mohorić and Takšić 2016) and the Analysis of Emotions Test (r = 0.46, *p* < .001). Moreover, analyses indicated that 44% of the VET’s variance is specific and not explained by general vocabulary knowledge (Takšić et al. 2021; Takšić and Mohoric 2008). Additionally, the test exhibited strong reliability (α = 0.90; Takšić and Mohoric 2008). The Portuguese adaptation of the instrument (Costa et al. 2011) has shown similarly strong psychometric properties, with an appropriate item difficulty level for the VET (M = 0.55; SD = 0.22), an internal consistency value of 0.71, and evidence of differential validity, reflecting both gender and cultural differences—Portuguese students performed better than Croatian students, and girls performed better overall.

#### 2.2.2. Academic Achievement

Academic Achievement—Students’ academic achievement was determined based on the grades assigned by teachers throughout the secondary education cycle. These grades reflect multiple indicators of student’s performance and are expressed in a quantitative way. In this study, students’ academic achievement was evaluated based on the following indicators:

(i) Grade Point Average (GPA)—The students’ final grades (ranging from 0 to 20) were obtained at the end of the 10th academic year from the school’s official records. The GPA used in the study was calculated as the average of the students’ grades across all subjects taken. In this study, students’ 10th grade GPA had a mean of 14.71 (SD = 0.16).
(ii) Mathematics—The students’ final grades in mathematics (ranging from 0 to 20) for the 12th academic year was collected from the school’s official records. In this study, students’ average score in 12th-grade mathematics was 14.57 (SD = 0.19).
(iii) Portuguese—The students’ final grades in maternal language (ranging from 0 to 20) for the 12th academic year was also collected from the school’s official records. In this study, the mean grade in 12th-grade Portuguese was 14.48 (SD = 0.18).

### 2.3. Procedure

The data were gathered through collective administration sessions in the classroom, attended by one of the researchers and the class teacher. Each student individually completed paper-based questionnaires following a brief group introduction about the study’s objectives and response instructions. Participants were informed that their participation was voluntary, their responses would remain confidential, and that choosing not to participate would have no impact. Minors were asked to obtain parental consent before participating, while students of legal age (18 years old) provided their own consent. The study adhered to ethical guidelines for research involving human participants, in accordance with the Declaration of Helsinki, and received approval from the Portuguese National Data Protection Commission, the Directorate General of Education, and the Faculty’s Ethics Committee.

### 2.4. Data Analysis

Longitudinal latent growth curve modeling (LGCM) was employed in the present study to explore how changes over time in the ‘understanding emotion’ dimension of EI relate to factors such as gender and GPA at the onset of secondary school, and to assess its influence on students’ academic outcomes (mathematics and maternal language) by the end of the school cycle. The latent growth curve analysis proceeded in two phases and was conducted using AMOS 28.0, applying maximum likelihood estimation. First, the study examined the changes in participants’ emotional understanding over the three years using an unconditional growth curve model, focusing on the initial status and rate of change (referred to as a ‘within-person’ model). The model was then enhanced by introducing predictor variables (the conditional model 1, i.e., a ‘between-person’ model). In the next phase, outcome variables were added to the model (the conditional model 2). The model’s goodness of fit was evaluated using the following criteria (Hu and Bentler 1999): Chi-square statistics, root mean square error of approximation (RMSEA) of 0.06 or less, comparative fit index (CFI), and Tucker–Lewis’s index (TLI, or non-normed fit index: NNFI), with values above 0.95 indicating a good fit.

## 3. Results

The analysis of skewness and kurtosis values confirmed the univariate normal distribution of the variables studied (both lower than 3.0 and 8.0, respectively; Kline 2005). Specifically, the skewness ranged from −1.062 (VET 12th grade) to 0.209 (Math’s 12th grade), while kurtosis values ranged from −0.903 (Math’s 12th grade) to 2.293 (VET 12th grade).

The descriptive analyses of the variables in the study are presented in Table 1. In general, the VET values demonstrated strong correlations across all three years of secondary school. VET values were closely linked to students’ GPA in the 10th grade and maternal language and mathematics grades. The most notable correlations were between students’ GPA in 10th grade and their final grades in mathematics and Portuguese. Gender was significantly associated with academic performance in 12th grade, with girls showing higher scores in both areas.

Descriptive statistics were conducted for each year to assess changes in emotional understanding levels over the three-year period (see Table 2). The results revealed a significant increase in VET levels from 10th to 12th grade, indicating a developmental trend. This finding justifies the use of longitudinal models, such as latent growth curve analyses, to further explore individual trajectories of change over time.

### 3.1. Individual Trajectories

**Figure 1:**
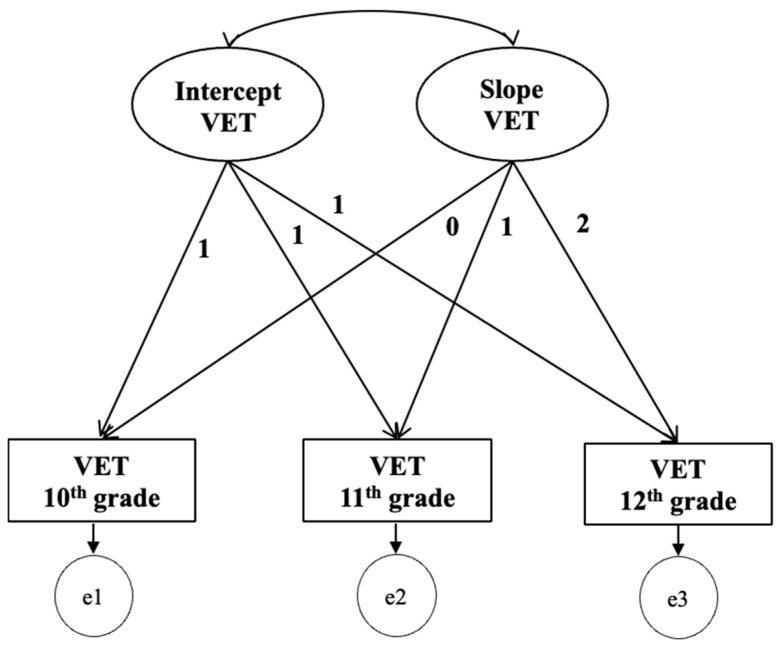
Unconditional latent growth curve model for intraindividual variability of the VET.

The LGC model identified the path of change in VET over the three years of secondary school (grades 10th, 11th, and 12th). An unconditional linear growth model was estimated for the EI understanding emotion domain (Figure 1), with intercept factor loadings constrained to 1 and slope factor loadings constrained to 0, 1, and 2.

Model M1 had a very good fit, *X*^2^ (8) = 1.93; *p* = .165, CFI = 0.996, RMSEA (HI90) = 0.06 (0.20). The VET intercept was significant and indicated the initial students’ VET level in the 1st year of secondary school, where *M* (VET intercept) = 22.35 (*SE* = 0.28; *p* < .001). The VET slope that indicated the VET rate of change across time was significant, with *M* (VET slope) = 0.95 (*SE* = 0.12; *p* < .001), indicating students’ VET levels increase throughout secondary school. The correlation between the VET intercept and slope was non-significant (*r* = −0.460, *p* = .177), which means that the initial levels of VET at 10th grade did not predict the students’ VET rate of change.

### 3.2. Predictors of EI Mindset Individual Trajectories

A conditional linear growth model was specified to test for gender and GPA predictors in the developmental trajectories of understanding emotion. The LGM conditioned on gender and GPA also had satisfactory fit, where *X*^2^ (4) = 5.60; *p* = .231, CFI = 0.995, RMSEA (HI90) = 0.04 (0.12). The results showed that gender did not predict the initial values of students’ understanding emotion (*β* gender.intercept = 0.383; *p* = .448). However, it did have an impact on students’ understanding emotion growth rate (*β* gender.slope = −0.587; *p* = .011), which provided evidence of different trajectories of development based on students’ gender; in this case, it indicated that girls developed their emotional understanding skills more during the secondary school cycle. GPA significantly and positively predicted the baseline values of VET (*β* GPA.intercept = 0.818; *p* < .001), which indicated that students with higher levels of GPA had higher initial levels of understanding emotion abilities, yet no effect was found in the rate of growth of VET (*β* GPA.slope = −0.069; *p* = .164).

### 3.3. Outcomes of EI Mindset Individual Trajectories

**Figure 2:**
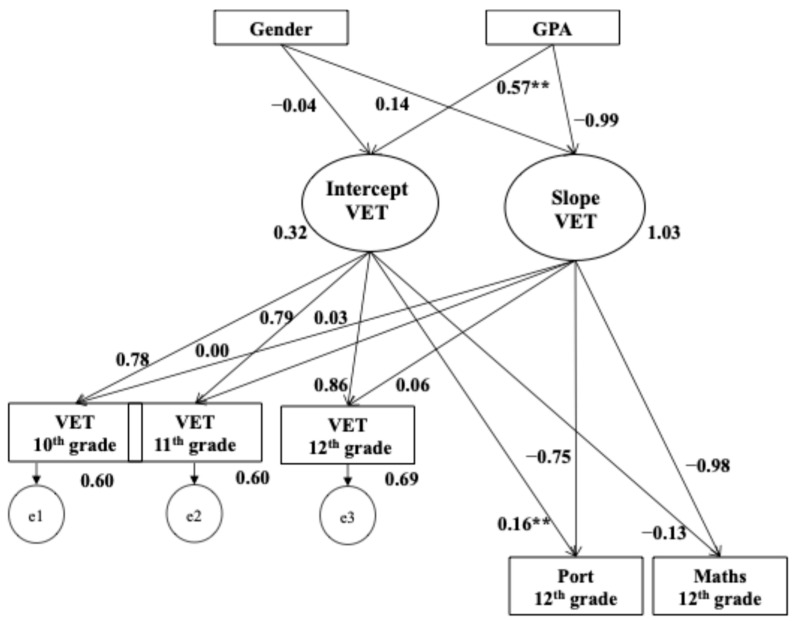
Conditional latent growth curve model for intraindividual variability of understanding emotion variables explained by the predictors (gender and GPA 10th grade) and outcomes (Maths and Portuguese language 12th grade); standardized coefficients are reported; ** *p* < .001.

Another conditional linear growth model was estimated to test for developmental trajectories VET on student’s academic outcomes (Maths and Portuguese) at the end of secondary school cycle (12th grade; cf. Figure 2). Thus, a new LGM model conditioned on gender—with GPA for 10th grade as a predictor—and on Maths and Portuguese marks in 12th grade was estimated. The model provided satisfactory fit, where *X*^2^ (12) = 24.35; *p* < .018, CFI = 0.982, RMSEA (HI90) = 0.07 (0.10).

Findings from the conditional model indicated that initial levels of VET in 10th grade (intercept) did not significantly predict students’ academic achievement in mathematics by 12th grade (β = −0.15, *p* = .142). However, initial VET levels were a significant positive predictor of achievement in the maternal language (*β* = 0.13, *p* < .001). On the other hand, the levels of understanding emotions throughout secondary school did not predict any of the academic outcomes in 12th grade.

## 4. Discussion

The current study aimed to extend the empirical understanding of the role of emotional skills on the students’ academic endeavors by examining the developmental trajectories of emotion understanding ability throughout the secondary cycle and their predictive value over students’ academic outcomes at the end of this cycle. Moreover, exploring students’ gender and prior academic achievement enabled further analyses of their potential effects on the developmental trajectories of emotional understanding and their impact over time.

Findings from this study revealed that the ability-based skill of understanding emotions tends to improve throughout secondary school. These results are consistent with the ability model of EI, which proposes that EI develops naturally with age, like other forms of intelligence (Mayer et al. 1999; Mayer and Salovey 1997). The literature further supports this view, indicating that both cognitive development as well as emotional and social experiences over time contribute to this progression (Brackett et al. 2016; Cabello et al. 2016; Navarro-Bravo et al. 2019). Additionally, previous empirical studies have confirmed that EI evolves during adolescence, with growth observed in the emotional understanding branch (Costa and Faria 2016; Megías-Robles et al. 2024). Simultaneously, the school environment, particularly during secondary school, becomes more demanding on cognitive, academic, and social levels, requiring students to develop emotional coping strategies and resources. Thus, this developmental stage appears to play a pivotal role in students’ emotional learning, particularly in experiential and more complex EI skills such as understanding emotions—as evidenced by the findings of this study.

### 4.1. Predictors and Outcomes of Emotional Understanding Developmental Path

Additionally, the effect of different predictors was explored considering the possibility of affecting the developmental trajectories of understanding emotions throughout the secondary school cycle. Gender was found to have an impact on understanding emotion’s ability development during late adolescence. In this case, the results revealed that girls tend to develop a greater ability to understand emotions compared to boys. These results may be influenced by the acculturation of female roles in society, where women are often more encouraged and expected to engage in emotional expression and processing (Brody and Hall 2008; Chaplin and Aldao 2013), in addition to inherent biological differences (Chaplin and Aldao 2013). Also, some studies have highlighted that boys tend to strengthen their emotional skills from young adulthood to middle age (Cabello et al. 2016). Indeed, gender differences have been extensively explored in the literature with different insights concerning both the different EI conceptualizations and assessment. Nonetheless, for ability-based EI studies, girls tend to outperform boys in different tasks (Megías-Robles et al. 2024).

Prior academic achievement has also been explored as a predictor for understanding emotion development throughout secondary school. Findings indicated that students’ academic achievement levels had no impact on the development of understanding of emotion skills, which means students with different academic achievement profiles may develop their emotional ability in a similar way. Therefore, although emotional competencies are associated with academic achievement (MacCann et al. 2020), this indicator does not appear to distinguish the progression of the emotional understanding skill across students with diverse profiles. It is plausible that the range of personal, social, and academic experiences typically encountered during late adolescence—particularly within a critical stage of educational development—contributes to more homogeneous opportunities for emotional learning. Furthermore, in the context of our sample, the secondary school students may represent a more academically selected subgroup. Since only those who progressed consistently over the three-year period were included in the follow-up, the resulting sample may exhibit reduced academic variability and influence the results. On the other hand, it was also found that at the beginning of secondary school, students with better academic achievement levels had better emotional understanding skills. Indeed, as expected according to the empirical evidence in the field, students with higher levels of emotional abilities tend to be more motivated to learn, to cope better and deal more effectively with stressful situations and, therefore, be more resilient, as well as having more positive relationships with peers and teachers, thus positively affecting academic outcomes (Gavín-Chocano et al. 2022; MacCann et al. 2020; Nieto et al. 2024; Sánchez-Álvarez et al. 2015; Tam et al. 2021).

This study investigated the effect of understanding emotions’ ability on students’ academic achievement at the end of secondary education. Students that better understand emotions at the beginning of the cycle attained, in this study, better academic achievement in Portuguese language subjects. These findings are consistent with the extensive literature that supports the positive link between EI, a more strongly EI ability-based model, and academic achievement (MacCann et al. 2020). Moreover, this link appears to be stronger when it comes to Portuguese, the participants’ maternal language. As highlighted by MacCann et al.’s 2020 meta-analysis, EI seems to play a significant role in academic performance, particularly in subjects that involve emotional content and personal experiences, such as those in the humanities domain. In these contexts, students are likely to express themselves more effectively and interpret emotional information more accurately in their native language, where they are more attuned to emotional nuances and better able to grasp the complexity and dynamics of emotional processing. Additionally, this result may also be influenced by a methodological aspect of the study. Since the measure used to assess emotional understanding was a verbal task involving emotional content, it is possible that students with stronger verbal skills were better equipped to perform well, thereby achieving higher scores in emotional understanding.

Although the literature has supported the positive link between EI abilities and academic achievement (MacCann et al. 2020), in this study, understanding emotions’ ability did not predict students’ mathematics grades at the end of the cycle. This result may be explained by the fact that students with a strong emotional orientation and socio-emotional competencies may find fewer opportunities to express or apply these competencies in abstract and impersonal subjects such as mathematics, whereas humanities subjects may offer greater scope for the integration and reflection of emotional experiences, competencies, and interpretations.

Moreover, the findings evidenced that the developmental trajectory of ability-based emotional understanding did not significantly predict students’ academic achievement at the end of the secondary cycle. Specifically, the rate of change in emotional understanding over time showed no significant association with final academic outcomes across subjects. This suggests that, in this study, improvements in emotional understanding throughout adolescence did not have a direct impact on academic performance. These results may indicate that the influence of these specific emotional skills on achievement is more nuanced, potentially mediated by other cognitive, motivational, or contextual variables or confounded by halo effects with biased evaluative judgments. On the other hand, the fact that the students’ prior academic achievement profiles in this study did not imply different rate changes in emotional understanding may consubstantiate this result, supporting that students’ emotional understanding might influence academic achievement mainly indirectly.

### 4.2. Limitations

The findings of this study should be interpreted considering its limitations. While this study acknowledges that various emotional abilities contribute to academic success, it focuses specifically on the ‘emotional understanding’ one. This decision was based on its potential relevance to younger students, considering its complexity and the absence of objective measures of EI, adapted to collective administrations and less time-consuming, particularly within the Portuguese cultural context. Moreover, the authors acknowledged that including a measure of students’ cognitive ability as a control variable in the longitudinal models would have been highly valuable, as it could help to disentangle the specific variability in the development of the EI ability of understanding emotions from potential cognitive growth over time. However, this measure was not collected as part of the longitudinal study and, therefore, could not be included as a control in the analyses. Considering the methodology of the study, another limitation is related to the sample, which limits the generalizability of the results. While sample attrition is common in longitudinal studies, the dropout rate in this study was significant. To address this, only participants who completed all three waves of data collection were included in the analyses, which consequently limited the sample size of the study. One key limitation is the relatively small number of measurement points, with only three data collection periods corresponding to each year of secondary school. A larger number of observations could have provided more detailed insights into the developmental trajectories of the emotional understanding domain, enabling more nuanced comparative analyses. Similarly, other variables could have been incorporated into the conditional models, either as predictors (e.g., cognitive ability, type of academic program, socioeconomic status) or outcomes (e.g., grades in science, arts, or sports, school attendance, engagement, well-being indicators, or academic feedback from teachers). Thus, future research should validate the present findings and further deepen the understanding in this area, particularly by examining the influence of additional EI abilities on students’ academic performance.

## 5. Conclusions

This study explored how students’ ability to understand emotions developed throughout Portuguese secondary education and investigated how this ability—along with gender and prior academic achievement—could predict later academic outcomes, including math, and maternal language achievement. Results indicated that the ability to understand emotions improved over time, with a steeper increase observed among girls. Furthermore, students’ emotional understanding at the beginning of secondary school predicted academic outcomes at the end of the cycle, although the trajectory of this development itself did not.

This study contributes to the less explored area of EI development during late adolescence, highlighting its impact on key academic outcomes such as academic achievement. Understanding these dynamics can help educators design targeted efforts and interventions to foster both students’ emotional abilities and their academic performance.

## Figures and Tables

**Table 1:** Descriptive statistics and zero-order correlations for all measures.

Measure	M(SD)	1	2	3	4	5	6
1. VET 10th	22.44 (0.29)	1					
2. VET 11th	23.09 (0.28)	0.64 *	1				
3. VET 12th	24.31 (0.25)	0.63 *	0.65 *	1			
4.GPA 10th	14.71 (0.16)	0.47 *	0.36 *	0.46 *	1		
5. Maths 12th	14.57 (0.19)	0.33 *	0.35 *	0.32 *	0.69 *	1	
6. Port 12th	14.48 (0.18)	0.52 *	0.40 *	0.46 *	0.85 *	0.62 *	1
7. Gender (0 = female; 1 = male)	-	0.02	−0.03	−0.13	−0.06	−0.07	−0.13

Note: VET—Vocabulary of Emotions Test; GPA = Grade Point Average; Maths = Maths grade; Port = Portuguese language *grade*. N = 222; Note. Bonferroni-corrected significance threshold set at *p* < .0018 based on 28 comparisons. * *p* < .0018.

**Table 2:** Descriptive statistics of VET levels for students by secondary school grade and paired t-test results.

	10th Grade			11th Grade			12th Grade			*t*-Tests (*df*)		
	*M*	(*SD*)	N	*M*	(*SD*)	N	*M*	(*SD*)	N	10th–11th grade	11th–12th grade	10th–12th grade
VET	22.44	4.27	222	23.09	4.11	222	24.31	3.69	222	−2.74 * (221)	−5.49 * (221)	−7.99 * (221)

Note: VET—Vocabulary of Emotions Test; *M* = mean; *SD* = standard deviation; *t*-tests = paired samples *t*-test score, paired samples *t*-tests were conducted to compare scores across timepoints. Results were evaluated using Bonferroni-corrected critical values (*t*_critical = 2.414 for *df* = 522, α_adjusted = 0.016). * *p* < .016.

## Data Availability

The data that support the findings of this study are available from the corresponding author upon reasonable request.

## Abbreviations

EI	Emotional Intelligence
GPA	Grade Point Average
LGC	Latent Growth Curve
VET	Vocabulary Emotions Test
